# Joint pausiness in parallel spike trains

**DOI:** 10.1186/1471-2202-16-S1-P218

**Published:** 2015-12-18

**Authors:** Matthias Gärtner, Sevil Duvarci, Jochen Roeper, Gaby Schneider

**Affiliations:** 1 Institute for Mathematics, Goethe-University, Frankfurt, Germany; 2 Neuroscience Center, Institute of Neurophysiology, Goethe-University, Frankfurt, Germany

## 

So-called 'pauses', i.e., periods with surprisingly few spikes, have recently gained increasing attention in the analysis of parallel spike trains of dopaminergic (DA) and Purkinje cells, in particular concerning simultaneity of pausing activity. The analysis of simultaneous pauses is usually based on the pauses identified in the separate spike trains. As a consequence, such techniques can suffer from the local definition of a pause within one spike train and can thus fail to identify joint pauses across spike trains that are easily detectable by eye. In addition, they crucially depend on the algorithm used for pause detection.

In order to tackle this problem, we present a new statistical method for the detection of synchronous pauses that focuses on typical characteristics of time periods showing synchronous pauses in parallel spike trains, and introduce a new measure for synchronous pausiness in parallel spike trains. We apply the technique to a data set of parallel DA neurons recorded from the VTA in freely moving mice. Interestingly, pausiness can be significantly increased in parallel spike trains as compared to individual processes or processes shifted by small time lags. This observation is robust and practically independent from the algorithm used for pause detection.

